# Paternal Inheritance of Mitochondrial DNA May Lead to Dioecy in Conifers

**DOI:** 10.1007/s10441-024-09481-1

**Published:** 2024-06-13

**Authors:** Tom J. de Jong, Avi Shmida

**Affiliations:** 1 Leiden University, PO Box 9505, 2300RA Leiden, The Netherlands; 2 Department of Ecology and Evolution and Center for the Study of Rationality, The Hebrew University of Jerusalem, Jerusalem, Israel

**Keywords:** Gymnosperms, Sex-allocation theory, Uniparental inheritance, Mitochondrion, Genomic conflict, Androdioecy

## Abstract

In angiosperms cytoplasmic DNA is typically passed on maternally through ovules. Genes in the mtDNA may cause male sterility. When male-sterile (female) cytotypes produce more seeds than cosexuals, they pass on more copies of their mtDNA and will co-occur with cosexuals with a neutral cytotype. Cytoplasmic gynodioecy is a well-known phenomenon in angiosperms, both in wild and crop plants. In some conifer families (e.g. Pinaceae) mitochondria are also maternally inherited. However in some other families (e.g. Taxaceae and Cupressaceae) mtDNA is paternally inherited through the pollen. With paternal mtDNA inheritance, male cytotypes that produce more pollen than cosexuals are expected to co-occur with cosexuals. This is uncharted territory. An ESS model shows that the presence of male cytotypes selects for more female allocation in the cosexual, i.e. for sexual specialisation. An allele that switches sex from male to female can then invade. This leads to rapid loss of the neutral cytotype of the cosexual, fixation of the male cytotype and dioecy with 50% males and 50% females. The models suggest that paternal inheritance of mtDNA facilitates the evolution dioecy. Consistent with this hypothesis the Pinaceae are 100% monoecious, while dioecy is common in the Taxaceae family and in the genus *Juniperus* (Cupressaceae). However, no reliable data are yet available on both mode of inheritance of mtDNA and gender variation of the same species. When cosexuals benefit from reproductive assurance (high selfing rate, low inbreeding depression, low fertilisation) they maintain themselves next to males and females. This predicted pattern with three sex types present in the same population is observed in conifers in nature.

## Introduction

**Fig. 1:**
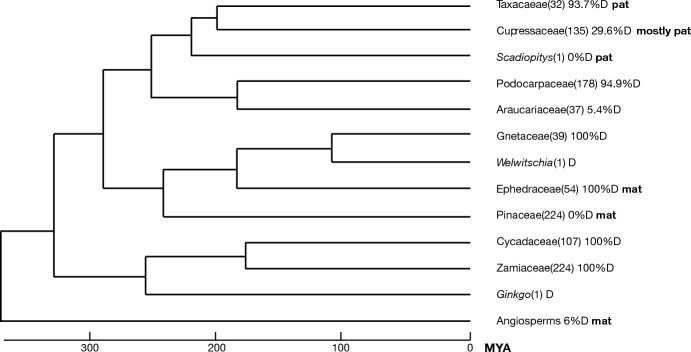
Phylogeny of gymnosperms (after Ran et al. 2018), indicating the percentage species that is dioecious (D) for different families (Walas et al. 2018) and the mode of inheritance of mtDNA (*mat* maternal, *pat *paternal, references in text). Number of species per family between brackets. *MYA* million years ago

About half (48%) of the 702 extant conifer species is dioecious, i.e. with separate pollen producing (male) and seed producing (female) individuals (Walas et al. 2018). The other half of the conifers is listed as monoecious, i.e. producing both male and female cones on the same individual. The frequency of dioecy in angiosperms (c.6% Renner and Ricklefs 1995) is much lower. Conifers may perhaps best be compared to wind-pollinated, monoecious shrubs and trees in the angiosperms. This group also has a lower incidence of dioecy (35.6% in the Israeli and 22.6% in the Dutch flora, de Jong et al. 2008) than conifers. Many angiosperm families contain some dioecious members (Dufay et al. 2014). The gymnosperms, consisting of cycads and conifers, are more uniform (Fig. 1). The two cycad families (331 species in total) are fully dioecious. For conifers Leslie et al. (2013) estimated that the transition to dioecy occurred between 10 and 13 times and the shift back to the ancestral monoecious stage between 6 and 9 times. Conifer families with close to 100% dioecious species include the Ephedraceae, Taxaceae, Gnetaceae and the large Podocarpaceae family. The relict species *Ginkgo biloba* and *Welwitschia mirabilis* are dioecious. In these strictly dioecious families monoecious individuals are rarely, if ever, observed. Contrary to this, all species in the Pinacaea, the largest conifer family, are monoecious. In the Araucariaceae family 35 out of 37 species are monoecious and these all grow in South-East Asia, Australia and Oceania. The two South American members of this family, *Araucaria angustifolia* and *A. araucana* (monkey-puzzle tree), diverged from their closest Australian relative about 30 million years ago (Kranitz et al. 2014) and are both dioecious. The large Cupressaceae family contains 5 fully dioecious and 24 fully monoecious genera (Walas et al. 2018). Only *Juniperus* contains a mix of dioecious and monoecious species is therefore the only conifer genus that allows a comparison of closely related species with a different sex system (Leslie et al. 2013). In *Juniperus* dioecy is the ancestral condition and monoecy evolved on five separate occasions in the section Sabina (Adams 2018). The frequency and distribution of dioecy over families is so different between gymnosperms and angiosperms that it demands an evolutionary explanation.

The main theoretical explanation for dioecy in angiosperms emphasises the outcrossing advantage of individuals with separate sexes, which avoids the production of selfed offspring of inferior quality (Charlesworth and Charlesworth 1981). If this is the case one expects dioecy to evolve in self-compatible species that cannot avoid selfing and not in self-incompatible species that are already fully outcrossing. This pattern indeed exists in angiosperms (Charlesworth 1985). Several authors (for instance, Bawa 1980; Charlesworth 1985) emphasised that ecological factors could also play a role. These factors could affect fitness directly or indirectly, through selfing and inbreeding depression. In angiosperms dioecy is positively associated with monoecy, abiotic pollination and a climbing growth habit (Renner and Ricklefs 1995).

Conifers do not easily fit this theory. Conifers are self-compatible (Hagman 1975) shrubs or trees with generally high (0.83–0.98) inbreeding depression (Sorensen 1999). Monoecious species already have high outcrossing rates, between 0.72–1.00 (Mitton and Williams 2006), that are apparently attained by separation of male and female cones in space and time and/or high rates of selective seed abortion (Neale and Wheeler 2019). Dioecy would increase outcrossing to 100%. However, as Givnish (1980) emphasised, a small increase in outcrossing rate is unlikely to provide a large benefit, especially since dioecy comes at a cost. Dioecy results in a loss of reproductive assurance and a lower ability to colonise new habitats (Pannell 2002; Friedman and Barrett 2009; Walas et al. 2018). Muñoz-Reinoso (2018) documented reproductive assurance in dioecious *Juniperus oxycedrus*:* a* rare isolated monoecious individual produced 42% more seeds than females of the same species. Givnish (1980) suggested that birds remove a greater fraction of the fleshy seeds on plants with many seeds. An accelerating female fitness gain curve could lead to dioecy (Charnov 1982). However, data do not support the suggested mechanism (de Jong and Klinkhamer 2005, p.40). Leslie et al. (2013) found no association between dioecy and mode of seed dispersal (fleshy/ non-fleshy seeds) in conifers after taking phylogeny into account. Apparently no consistent associations between sex system (monoecy/dioecy) and plant characters have been reported in conifers. So how could one explain the marked differences between the families? In this paper we explore the hypothesis that paternal inheritance of cytoplasmic DNA facilitates the evolution of dioecy.

In angiosperms cytDNA is nearly always maternally inherited, i.e. through ovules produced on the mother plant and not through pollen (Mogensen 1996). Such uniparental inheritance results in genomic conflict over sex allocation. cytDNA is selected to bias sex allocation in such a way that more seeds are produced and more cytDNA copies are passed on to the next generation (Burt and Trivers 2006). Nuclear restorer genes are selected to neutralise these effects. Cytoplasmic Male Sterility (CMS) has been reported in many angiosperms, both in wild and crop plants (Budar et al. 2003). Plants with the male-sterile cytoplasm produce dysfunctional pollen and show a distinct phenotype with aborted stamens and/or empty pollen grains. These male-sterile, and therefore functionally female, cytotypes coexist with cosexuals (gynodioecy). The genetic mechanisms behind male sterility have been extensively studied in angiosperms. Budar et al. (2003) suggested an active role for mitochondria during male gametogenesis. A mutation leading to loss of function would then result in sterile pollen being produced. In angiosperms few studies pointed to a role of the chloroplast in inducing CMS, but examples do exist (for instance, van der Hulst et al. 2004).

Both male and female sterility have been reported in conifers (references in Fritsche et al. 2018) but the relation to cytoplasmic factors has not been studied. Male sterility was reported to be rare in a detailed study by Moriguchi et al. (2020) of natural populations of *Cryptomeria japonica.* However it is not so easy to climb up a tree and inspect male cones with a loupe, so the presence of sterile pollen may have be underestimated in other species. Interestingly, several authors (Marmi et al. 2023 and references therein) mentioned male sterility as a possible cause for extinction of conifer species, due to the absence of pollen in the fossil remains of their last remaining populations. Unlike angiosperms with perfect, hermaphrodite flowers, the cones initiated by gymnosperms are either male or female. This opens the possibility that the cytoplasm could affect the gender of the cone at initiation, for instance through hormones. This mechanism would operate at the earliest stage of cone formation and would be more efficient in redirecting resources from male to female or vice versa, than abortion at a later stage. Unlike male sterility this mechanism would not be immediately visible from the phenotype and could only be revealed by making crosses (see paragraph 8.3). We use the terms “female” and “male” for gymnosperms rather than “male-sterile” and “female-sterile” as is common in the literature on angiosperms. We use cosexual as a general term for species in which individuals produce both pollen and seeds.

If maternal inheritance of cytDNA selects for a female bias and gynodioecy, then paternal inheritance of cytDNA should select for a male bias and populations consisting of male cytotypes and cosexuals (androdioecy, Burt and Trivers 2006). Paternal inheritance of cytDNA is very rare among angiosperms but common among gymnosperms (Mogensen 1996). With the exception of the Ephedraceae, in conifers cpDNA is generally inherited through pollen (Mogensen 1996). The mode of mitochondrial inheritance varies. In the Pinaceae genetic data on 8 species (including the genera *Pinus*, *Picea*, *Larix* and *Pseudotsuga*) show maternal inheritance of mtDNA (Adams 2019). This was confirmed by studies on genetic structure of populations using cpDNA and mtDNA markers for 16 other species in the Pinaceae (Bagnoli et al. 2011). Recent genetic studies showed paternal inheritance of mtDNA in the Taxaceae (Chybicki et al. 2016; Su et al. 2018) and in the Japanese endemic *Scadiopitys verticillata*, the sole member of the Scadiopitaceae family (Worth et al. 2014). In the Cupressaceae the mitochondrion was paternally inherited in 3 *Juniperus* species and in 5 additional species from other genera, while in 2 species maternal inheritance was suggested (Mogensen 1996; Adams 2019). Mogensen (1996) concluded that mtDNA inheritance was maternal in the Ephedraceae based on a microscopic study. For other families there are just too few data to reliably complete Fig. 1. Figure 1 suggests differences between gymnosperm families in mode of mtDNA inheritance that are highly conserved through evolution.

In this paper we develop the hypothesis that paternal inheritance of cytDNA facilitates the evolution of dioecy. First we outline models for calculating Evolutionarily Stable Strategies (ESS) for sex allocation in outcrossing and partially selfing plants. Results of ESS models are generally the same as those from specific genetic models for diploids and haploids (van Cleve 2023). The advantage of the ESS models is that they are simpler, allow more insight in the result and tool kits for analysis are available (Geritz et al. 1998). Next we calculate frequencies of female and male cytotypes in populations of cosexuals. We then ask how the presence of these cytotypes affects sex allocation of the cosexual. Sexual specialisation only occurs in response to the presence of male cytotypes. With genetic models we illustrate how a system with males and female-biased cosexuals could evolve to dioecy and, more generally, how paternal inheritance of male cytotypes affects the sex system of conifers.

## Sex Allocation Models

We introduce the simplest ESS model for relative allocation to male and female in outcrossing plants, using the Shaw-Mohler equation to estimate male success. We then extend this model for partially selfing plants with some variations. Selfing rate could be constant or an increasing function of male allocation. Fertilisation could be constant or increase with amount of pollen in the population. For all cases we calculate the ESS for sex allocation of a cosexual.

### Assumptions

This approach builds upon the ESS model that was introduced for selfing plants by Charlesworth and Charlesworth (1981). Plants allocate a fraction *r* of their resources to male cones with pollen. Due to a trade off, the remainder of the resources, 1-*r*, is allocated to female function (full compensation). Female allocation begins with making female cones that contain rarely one and usually more female gametophytes that develop in ovules that each contain one egg cell. After these egg cells have been fertilised the developing diploid seed, the sporophyte, is nourished by the mother plant. These costs also accrue to female allocation. Conifer seeds develop over an extended period that could last months or years and over this period the seed producing cone extends in size. It is assumed that with full fertilisation a constant fraction of the investment in female is allocated to supporting structures, the seed cone, and the remainder is allocated to seeds, so that number of seeds produced is proportional to the investment $1-r$. Costs of female cones that are unfertilised or aborted (see point *ii* below) are also part of female allocation. Sex allocation is dictated by nuclear alleles and *r* can take on any value between 0 and 1. With *r* = 0 the plants is female, with *r* = 1 it is male and at intermediate values it is cosexual. Partly selfing cosexual plants transfer nuclear genes to the next generation by three routes: (*i*) selfed seeds, (*ii*) outcrossed seeds and (*iii*) siring seeds on other plants in the population.

(*i*) The selfing rate is denoted as *S*, a fraction $1-S$ of the seeds is outcrossed ($0\le S\le 1$*)*. Self-pollination is always possible and provides reproductive assurance. Selfed seeds suffer from inbreeding depression, the relative viability of a selfed seed compared to an outcrossed seed is $1-\delta$, with $0\le \delta <1$. A high value of $\delta$ corresponds to high inbreeding depression, i.e. low offspring viability. Selfed seeds contain 2 allele copies, the plant is both the mother and the father. It is assumed that prior selfing does not reduce the amount of pollen available for outcrossing. For wind-pollinated plants it seems reasonable not to discount the grains used in selfing with pollen export. Pollen grains deposited on the bracts of female cones on the same plant are lost anyway, whether they fertilise a nearby ovule or not. The selfing rate is assumed constant, independent of male allocation. This would be expected when self pollen saturates ovules in nearby female cones. For instance, in *Cedrus* male and female cones can be next to each other on the same branch. A single male cones release in the order of 10^5^ to 10^6^ pollen grains, more than enough to fertilise all egg cells in the adjacent female cone. Higher male allocation, more pollen grains per cone or making more male cones elsewhere on the plant, would not matter for the selfing rate. Ferriol et al. (2011) found no relation between the highly variable male cone production of a *Cedrus atlantica* tree and its selfing rate. In this case selfing rate may depend on synchrony of pollen release and ovules becoming available, for instance, through the presentation of a pollination droplet. Alternatively, the prior selfing rate increases with male allocation. When male and female cones are placed further apart, fewer self pollen grains land near ovules on the same plant and making some more pollen could increase prior selfing. Conifers produce so much pollen that we expect that even low values for male allocation (*r*) already lead to saturation of nearby ovules*,* in which case the selfing curve decelerates strongly with *r*. We briefly consider this alternative for a constant selfing rate in paragraph 2.4. In both cases we assume prior selfing: selfing comes first and self-fertilised eggs are no longer available to outcross pollen that arrives later. Competition for ovules between outcross and self pollen grains could be a relevant extension. We did not include this in the models but will briefly discuss this option.

*(ii)* Fertilisation with pollen from other plants is not assured and will depend on the amount of pollen floating in the air. We account for this by reducing fitness returns from female allocation by a factor *F (*$0\le F\le 1$*).* $F<1$ reflects costs of making supporting female structures that are later aborted or become useless when fertilisation does not take place. In gymnosperms the nutritive tissue in the seed (‘endosperm’) is haploid and develops from a female megaspore, independent from fertilisation. When fertilisation fails this investment is lost. In some cases, for instance in cycad genus *Encephalartos*, complete seeds are formed even without fertilisation. These seeds contain no embryo and are unviable. With poor fertilisation female cones reached a regular size at maturation with few (Goubitz et al. 2002) or even no seeds (Ortiz et al. 1998). Poor fertilisation therefore involves a cost, which is not necessarily small. We refer to *F* simply as fertilisation rate.

*(iii)* Pollen is well mixed in a large population and competes for the ovules that remain after prior selfing. Fitness returns from male investment depend on the ratio of outcrossed seeds to pollen in the population.

### Sex Allocation in Outcrossing Plants

We count from the moment seeds are produced to the same moment one generation later. Each seed contributes equally to the female component of fitness. This model assumes no acceleration or deceleration of the fitness gain curves due to ecological factors and fitness gain curves are linear in the sense of Charnov (1982). All common plants with sex-allocation strategy *r* allocate $1-r$ to female and seed production per plant is proportional to $F(1-r)$. Each seed receives one allele copy from the mother and one from the father. The number of allele copies successfully passed on to the next generation by the common type is:

1a $$W=2F(1-r)$$

A mutant allocates *r*_*m*_ to male and $1-{r}_{m}$ to female. In a large (*N* plants) well-mixed population the pollen from the mutant competes with $Nr$ pollen from the common type for $N(1-r)$ ovules. The expected number of seeds sired by a single mutant is: $N(1-r)({r}_{m}/Nr)=(1-r){r}_{m}/r$. Population size *N* cancels rom the equation. Absolute fitness of the rare mutant is the sum of seeds produced and those sired on other plants:

1b $${W}_{m}=F(1-{r}_{m})+F(1-r){r}_{m}/r$$

which is known as the Shaw-Mohler equation (Charnov 1982). Invasion fitness of the mutant is:

1c $${w}_{m}={W}_{m}-W$$

If ${w}_{m}>0$ the mutant can invade the population. The candidate ESS is found by differentiating ${w}_{m}$ with respect to *r*_*m*_ and setting the derivative equal to zero (Geritz et al. 1998). This yields $d{w}_{m}/d{r}_{m}=(1-2r)/r=0$ so that the candidate ESS is:

1d $$r^{*}=0.5$$

Outcrossing plants are selected to allocate equal amounts of resource to male and female. Does the population reach r*? This follows from ${d}^{2}{w}_{m}/d{r}^{2}>0$ (Geritz et al. 1998). Here ${d}^{2}{w}_{m}/d{r}^{2}=2F{r}_{m}{r}^{-3}$, which for ${r}_{m}=r=r^{*}$ becomes equal to $2F{r}^{-2}$ and this is always positive. Therefore the population converges to the candidate ESS r*. Does r* represents a fitness maximum or fitness minimum (Geritz et al. 1998)? With a fitness maximum, or strong ESS, an intermediate value of r* is stable*,* i.e. rare mutants with a slightly different strategy have lower fitness and cannot invade. A fitness minimum is a branching point, from which the population could possibly evolve to dioecy, a mix of individuals with $r=0$ and $r=1$ (Geritz and de Jong 2001). For Eq. (1c) ${d}^{2}{w}_{m}/d{{r}_{m}}^{2}=0$, which describes a so-called weak ESS. This applies when the fitness equation is linear in ${r}_{m}$, which is the case in Eq. (1b) and all fitness equations that follow (except those in paragraph 2.4). When the population has reached r*, all rare mutants have the same fitness as the common type and therefore cannot invade.

### Sex Allocation in Partially Selfing Plants

For plants that are both selfing (*S*) and outcrossing (1-*S*) the fitness equation of the mutant becomes:

2a $${W}_{m}=2(1-\delta )S(1-{r}_{m})+F(1-S)(1-{r}_{m})+F(1-S)(1-r){r}_{m}/r$$

The fitness *W* of the common type follows from setting ${r}_{m}=r$ in Eq. (2a) and again ${w}_{m}={W}_{m}-W$. This applies to all fitness equations that follow. The three terms in Eq. (2a) refer to selfed seeds produced (*i*), outcrossed seeds on the mother plant (*ii*) and seeds sired on other plants in the population (*iii*), respectively (Charlesworth and Charlesworth 1981). Factors $2(1-\delta )$ and *F* weigh fitness contributions of selfed and outcrossed seeds, respectively. Computing $d{w}_{m}/{r}_{m}=0$ gives the weak ESS to which the population converges:

2b $$r^{*}=\frac{F(1-S)}{2S(1-\delta )+2F(1-S)}$$

Values predicted by Eq. (2b) range between 0 and 0.5, so that selfing always results in female bias. $d{r}^{*}/dS \le 0$, more selfing always leads to a female-biased allocation ($r^{*}<0.5$). $dr^{*}/dF$ is positive. Improved fertilisation leads to more allocation to male and less to female. Under conditions of poor fertilisation plants are selected to allocate more to female. Selfed seeds can always be produced and give reproductive assurance when the chance of fertilisation is slim. This strategy of producing selfed seeds is especially successful with low inbreeding depression ($\delta$). In an outcrossing population (*S* = 0) the factor *F* cancels from Eq. (2b) and the ESS is again to allocate equal amounts of resource to male and female ($r^{*}=0.5$). With full fertilisation ($F=1$) Eq. (2b) reduces to the familiar (Charlesworth and Charlesworth 1981):

2c $$r^{*}=(1-S)/(2-2S\delta )$$

### Selfing Rating Increases with Male Allocation

We write the selfing rate of the mutant as *S*_*m*_ to indicate that selfing could increase with male allocation of the mutant. Fitness of a a rare mutant with strategy *r*_*m*_ is:

3a $${W}_{m}=2(1-\delta ){S}_{m}(1-{r}_{m})+F(1-{S}_{m})(1-{r}_{m})+F(1-S)(1-r)\frac{{r}_{m}}{r}$$

When selfing is some decelerating function of male allocation (like $S={r}^{\alpha }$ with$0<\alpha <1$) there is no simple equation for the candidate ESS. Still r* can always be calculated by comparing fitness of common type and mutant (Eq. 3a). In this version of the model selfing still selects for relatively more female allocation (de Jong et al. 1999) but results also depend on $d{S}_{m}/d{r}_{m}$ that can take on any (positive) value. This complicates the analysis. The population converges to the candidate ESS when ${d}^{2}{w}_{m}/d{r}^{2}>0$. Here${d}^{2}{w}_{m}/d{r}^{2}=2F(1-S){r}_{m}/{r}^{3}$, which for ${r}_{m}=r=r^{*}$ equals $2F(1-S)/{r}^{2}$ and this is indeed positive. The fitness maximum or minimum can be calculated from${d}^{2}{w}_{m}/d{r}_{m}^{2}$:

3b $${d}^{2}{w}_{m}/d{r}_{m}^{2}=[2(1-\delta )-F][{S}_{m}^{{^{\prime}}{^{\prime}}}(1-{r}_{m})-2{S}_{m}^{{^{\prime}}}]$$

in which ${S}_{m}^{{^{\prime}}}$ and ${S}_{m}^{{^{\prime}}{^{\prime}}}$ are the first and second derivative of *S* with respect to *r*_*m*_*,* respectively*.* With a constant selfing rate both ${S}_{m}^{{^{\prime}}}$ and ${S}_{m}^{{^{\prime}}{^{\prime}}}$ are zero, ${d}^{2}{w}_{m}/d{r}_{m}^{2}=0$ and r* is a weak ESS. When the selfing rate is an increasing and decelerating function of male allocation, *S’* is positive and *S*’’ is negative. The term on the right hand side is then negative and the sign of ${d}^{2}{w}_{m}/d{r}_{m}^{2}$ depends on the term $2(1-\delta )-F$ on the left hand side. The candidate ESS r* is a fitness maximum when ${d}^{2}{w}_{m}/d{r}_{m}^{2}$ is negative, i.e. when $\delta <1-F/2$. With $\delta <0.5$ cosexuality, an intermediate value of r*, is always an ESS. Low fertilisation rates stabilise cosexuality: with *F* = 0.5 cosexuality is selected when $\delta <0.75$. A fitness minimum, or branching point, exists when ${d}^{2}{w}_{m}/d{r}_{m}^{2}$ is positive, i.e. when $\delta >1-F/2$. The population could evolve to dioecy with full fertilisation (*F* = 1) and $\delta >0.5$. At lower fertilisation rates the conditions for branching become more restrictive.

### Fertilisation Increases with Male Allocation

Fertilisation rate may not be constant but may increase with the amount of pollen in the population. So far all plants are cosexual and produce *r* pollen. Fertilisation could then be estimated as $F={r}^{\alpha }$ in which we set $\alpha =1$ for convenience. With constant selfing rate the model becomes:

4a $${W}_{m}=2(1-\delta )S(1-{r}_{m})+r(1-S)(1-{r}_{m})+r(1-S)(1-r)\frac{{r}_{m}}{r}$$

$d{w}_{m}^{2}/{d}^{2}r=4(1-S)$ is positive so the population converges to the candidate ESS. The weak ESS is found by setting $d{w}_{m}/d{r}_{m}=0$ which gives:

4b $$r^{*}=0.5-0.5p(\delta ,S)$$

With $p(\delta ,S)=2(1-\delta )S/(1-S)$*.* The value of function $p(\delta ,S)$ declines with $\delta$ and increases with *S* and indicates how much fitness plants gain from making selfed seeds. To keep notation concise we sometimes use *p* for $p(\delta ,S)$ in what follows. In fully outcrossing populations ($S=0$), $p=0$ and $r^{*}=0.5$ is the EES*.* A combination of a high outcrossing rate and high inbreeding depression may be typical for conifers. For $S=0.2$ and $\delta =0.8$, $p=0.1$ and r* has an intermediate value (0.45). In mostly selfing populations with low inbreeding depression $p>1$. Allocation cannot take on a negative value, so with $p>1$ Eq. (4b) predicts that $r^{*}=0$ is the ESS. This is the case when $(3-2\delta )S \ge 1$. When cosexual plants allocate zero to male function they only gain fitness through selfed seeds. Such selection for producing as much selfed seeds as possible may occur in this model when fitness gains from selfing outweigh those from outcrossing (Charnov 1982).

## Frequency of Female and Male Cytotypes in a Cosexual Population

We calculate how many copies of the neutral, female and male cytoplasm are passed on to the next generation. Next we compute the equilibrium frequency for populations with cytoplasmic females and cosexuals (gynodioecy) and cytoplasmic males and cosexuals (androdioecy).

### Frequency of Female Cytotypes

#### Constant Fertilisation Rate

Equation 2a needs to be adapted for uniparental transfer. Only a single copy of the cytotype is passed on through selfed seeds, not two copies as for a nuclear allele when the plant is both the mother and father. With maternal transfer a neutral cytotype is passed on only through the selfed (*i*) and outcrossed seeds (*ii*). Hence ${W}_{cosexual}=(1-\delta )S(1-r)+F(1-S)(1-r)$. The female cytotype is passed on through outcrossed seeds only and since the females allocate nothing to male ($r=0$): ${W}_{female}=F$. In a fully outcrossing population the female always passes on more copies of her cytoplasm than the cosexual. With some selfing the female cytotype wins when: $F>(1-\delta )S(1-r)/(S-Sr+r)$. When *F* is below this threshold the neutral cytotype wins. The two cytotypes do not coexist.

#### Fertilisation Rate Decreases with Fraction Females in the Population

Fertilisation may not be constant but is likely to increase with the frequency of cosexuals (*g)* and how much pollen they produce: $F=(g{r})^{\alpha }$ with $0<\alpha\le 1$. With $\alpha =1$ this simplifies to $F=gr$. As females increase in frequency, their fertilisation rate decreases and seed production is reduced (Lewis 1941). Cosexuals enjoy reproductive assurance from their selfed seeds. In a partly selfing population a stable equilibrium $\tilde{g}$ is reached when females and cosexuals pass on the same number of copies of their cytoplasm (${W}_{cosexual}={W}_{female}$):

5 $$\tilde{g}=(1-\delta )S(1-r)/(Sr-S{r}^{2}+{r}^{2})$$

The equilibrium frequency of the female cytotype ($1-\tilde{g}$) increases with more male allocation in the cosexual ($d\tilde{g}/dr<0$). Equation 5 predicts values for $\tilde{g}$ that are greater than 1 when *r* is small. Of course, $\tilde{g}=1$ is the maximum value at which only cosexuals remain. When conditions favour the cosexual (low *r,* low $\delta$, high *S*), the female cytotype disappears from the population.

### Frequency of Male Cytotypes

#### Constant Fertilisation Rate

Consider a cytotype that is paternally transmitted through pollen and not through ovules. Cosexuals then pass on their neutral cytotype through selfing) (*i*) and by being the father of outcrossed seeds on other cosexual plants where their pollen competes against pollen from male cytotypes (*iii*). Males produce only pollen ($r=1$) and pass on their male cytotype by siring outcrossed seeds on cosexuals (*iii*) in competition with pollen from cosexuals. Cosexuals have frequency *g* and males frequency 1-*g*. The probability that a seed is sired by cosexuals equals the pollen they produce (*gr*) divided by pollen in the population $(gr+1-g)$. To calculate the probability that an individual cosexual plant is the father of the seed we need to divide by *g,* and this gives $r/(gr+1-g)$*.* Only cosexuals produce seeds so availability of outcrossed seeds in the population is reduced from $F(1-r)(1-S)$ with only cosexuals in the population (*g* = 1) to $gF(1-r)(1-S)$. The number of copies of its neutral cytotype that a cosexual individual passes on in a mixed population with cytoplasmic males is:

6a $${W}_{cosexual}=(1-\delta )S(1-r)+gF(1-r)(1-S)\frac{r}{gr+1-g}$$

The number of copies of the male cytotype that a male individual passes on is:

6b $${W}_{male}=gF(1-r)(1-S)\frac{1}{gr+1-g}$$

Rare males pass on many copies of their cytotype, but when their frequency increases, the frequency of the cosexual decreases and fewer ovules become available for pollen from males. Cosexuals enjoy reproductive assurance through their selfed seeds. In the stable equilibrium, with constant fertilisation rate *F*, the equilibrium frequency of cosexuals $\tilde{g}$ is:

6c $$\tilde{g}=\frac{(1-\delta )S}{(1-\delta )S(1-r)+F(1-S)(1-r)}$$

**Fig. 2:**
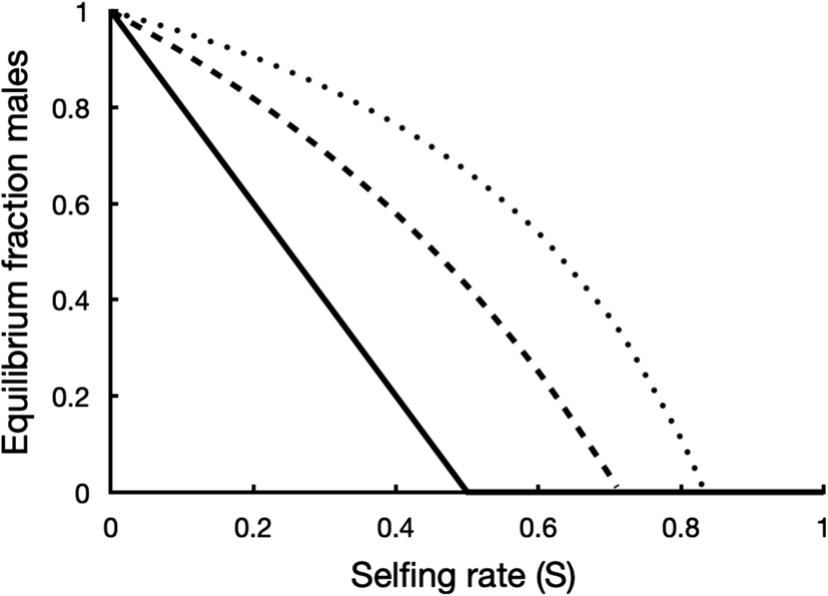
Equilibrium fraction cytoplasmic males (1-$\tilde{g}$*,* Eq. 6c) as a function of the selfing rate (*S*) in a mixed population with cosexuals. The fertilisation rate (*F*) is fixed. *F* = 0.2 (solid line), 0.5 (broken line) and 1 (stippled line). Cosexuals allocate equally to male and female (*r* = 0.5) and inbreeding depression ($\delta$) was set at 0.8

Males increase to 100% when *S* = 0, in which case the model population would go extinct.$d\tilde{g}/dS \ge 0$ and $d\tilde{g}/d\delta \le 0$*.* Cosexuals benefit most from reproductive assurance when a high selfing rate is combined with low inbreeding depression. In addition $d\tilde{g}/dr>0$, higher male allocation in the cosexual leads to more cosexuals and fewer males. Note that with males in the population the effect of increasing *r* on cosexual frequency is opposite to the same effect with females present (paragraph 3.1.2). Males need cosexuals to be able to sire seeds and low fertilisation and high selfing combined with high male allocation in the cosexual (fewer ovules available and more pollen competition) limit their mating success. At low selfing rates the two types coexist but the males disappear from the population at high selfing rates (Fig. 2). Males are lost from the population at $\tilde{g} \ge1$, so that Eq. (6c) can be rewritten as:

6d $${F}_{critical}\le \frac{r\left(1-\delta \right)S}{\left(1-r\right)\left(1-S\right)}$$

When the fertilisation rate is below this threshold males disappear from the population. The term on the right on the right hand side can take on values greater than one, in which case males are eliminated even with 100% fertilisation (*F* = 1).

#### Fertilisation Rate Increases with Pollen in the Population

Alternatively, one could assume that fertilisation is not constant but *F* increases with the total amount of pollen in the population. The cosexuals contribute *gr* and males 1-*g* to this amount. Therefore: $F=(gr+1-g{)}^{\alpha }$. With $\alpha =1$ this gives the equilibrium frequency of cosexuals:

7 $$\tilde{g}=\frac{(1-\delta )S}{(1-r)(1-S)}$$

which differs somewhat from Eq. (6c) but $\tilde{g}$ depends in a similar way on parameters *r, S* and $\delta$. Coexistence is possible but males are again eliminated when they have too few mating opportunities.

## Effects of Female Cytotypes on Sex Allocation of the Cosexual

We now calculate how a fixed fraction cytoplasmic females in the population would affect the ESS for sex allocation in the cosexual. These effects depend on whether fertilisation rate is constant or depends on the amount of pollen in the population.

### Constant Fertilisation Rate

**Fig. 3:**
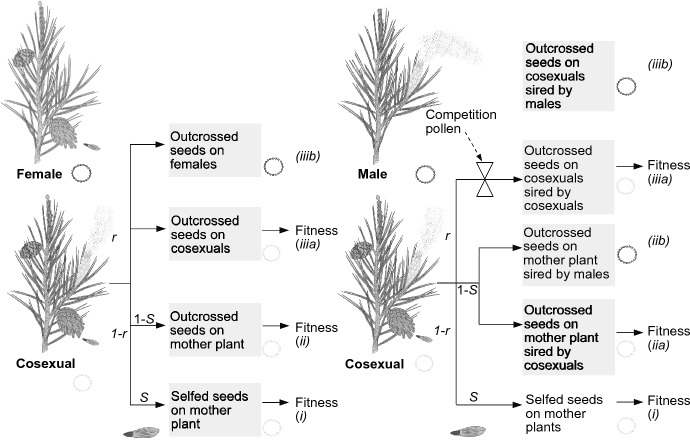
The effect of a female cytotype with maternal transmission (left) and a male cytotype with paternal transmission (right) on fitness of nuclear sex-allocation alleles in a cosexual plant. The circular cytDNA of the female or male (dark) and cosexual (light) is indicated. The scheme illustrates Eqs. (8) and (10) and indicates which routes contribute to fitness of a nuclear sex-allocation allele when the cosexual grows together with a cytoplasmic female or cytoplasmic male. The cosexual plant allocates a fraction *r* of its resource to male cones with pollen and 1-*r* to female cones with seeds. *S *selfing rate

One would intuitively expect that the presence of female cytotypes in the population leads to selection for a male bias in the cosexual. This is not the case, it could even be the opposite. Cosexuals pass on nuclear genes by producing selfed seeds (*i*) and by producing outcrossed seeds on the mother plant (*ii*) as before. Cosexuals also sire seeds on other cosexuals *(iii*a) and on female plants (*iii*b) (Fig. 3)*.* Selection on nuclear sex-allocation alleles occurs within the isolated compartment of the neutral cytotype. Nuclear genes from the cosexual that go through route (*iii*b), seeds sired on females, become permanently associated with the female cytotype. Females produce only females and once an allele is in a female it never returns to the neutral cytoplasm. Route *iii*a counts for fitness but route *iii*b is a sink and should be discarded for fitness. Without the females all cosexuals together gained $F(1-S)(1-r)$ fitness from siring seeds on other cosexuals. With females in the population only *g* cosexuals are left and this reduces the number of seeds that can be sired on other cosexuals to $gF(1-S)(1-r)$.

Does this matter for sex allocation of the cosexual? No, because only *g* cosexuals are left in the population and for the calculating male success per individual one needs to divide the success of all cosexuals by *g.* The two *g*’s cancel from the equation and with constant *S*:

8 $${W}_{m}=2(1-\delta )S(1-{r}_{m})+F(1-S)(1-{r}_{m})+(1/g)gF(1-S)(1-r)\frac{{r}_{m}}{r}$$

After crossing out the *g’s* Eq. (8) is similar to Eq. (3a). Sex allocation of the cosexual does not change with female cytotypes present in the population. This result also holds when the selfing rate is not constant and increases with male allocation (as in Eq. 3a). The result was already well-known from genetic models on the evolution of gynodioecy (for instance, Maurice et al. 1993, 1994).

### Fertilisation Rate Decreases with Frequency of Females

Alternatively, fertilisation decreases with the fraction females and therefore increases with the fraction cosexuals and how much pollen they produce: $F=(gr{)}^{\alpha }$. We assume $\alpha =1$ and write $gr$ for *F* in Eq. (3a) (or in Eq. 8). With constant *S* mutant fitness is:

9a $${W}_{m}=2(1-\delta )S(1-{r}_{m})+gr(1-S)(1-{r}_{m})+gr(1-S)(1-r)\frac{{r}_{m}}{r}$$

The population converges to the ESS r* because ${d}^{2}{w}_{m}/d{r}^{2}=4g(1-S)$ is positive. The ESS follows from $d{w}_{m}/d{r}_{m}=0$:

9b $$r^{*}=0.5-0.5p(\delta ,S)/g$$

This reduces for $g=1$ to Eq. (4b). A higher fraction cosexuals (*g*) leads to more male allocation (r*). Adding females to the population thus selects for more female allocation in the cosexual, i.e. the opposite of sexual specialisation. The new effect of females is that they reduce fertilisation rate from *r* with only cosexuals ($g=1$) to *gr* with a fraction $1-g$ females present (Eq. 9a). Fewer outcrossed seeds can be produced by the mother plant and fewer can be sired on other cosexuals (route *ii* and *iii* in Fig. 3). Adding female cytotypes to the population is similar to reducing *F* in the simpler Eq. (2a). The condition for an intermediate ESS ($0<r<1$) is now $g>p(\delta ,S)$. At high values of $p(\delta ,S)$*,* $r^{*}=0$ is predicted. The conditions for an intermediate ESS in Eq. (9b) are more restricted than those in Eq. (4b) (without the females). With $r^{*}=0$ cosexuals maintain themselves only through the selfed seeds they produce. These cosexuals no longer export pollen so females will not be fertilised and will disappear from the model population.

## Effects of Male Cytotypes on Sex Allocation of the Cosexual (With Constant Fertilisation)

We calculate how a fixed fraction cytoplasmic males in the population affects the ESS for sex allocation in the cosexual. First we sketch the rather complicated case where the selfing rate increases with male allocation. Second we continue with constant selfing and examine the ESS for sex-allocation when fertilisation rate is constant or when it increases with the total amount of pollen in the population, produced by both cosexuals and males.

Male cytotypes occur at frequency 1-*g* next to *g* cosexuals with a neutral cytotype*.* Nuclear alleles from the cosexual are passed on through (*i*) selfed seeds, (*ii*a) outcrossed seeds on the mother plant that are sired by another cosexual and (*iii*a) seeds sired on other cosexuals (Fig. 3). As in paragraph 4, selection on nuclear sex-allocation alleles occurs only in the isolated compartment of the neutral cytoplasm. The presence of the cytoplasmic males affects fitness of nuclear sex allocation alleles in the cosexual in two ways. First, the outcrossed seeds on the cosexual that are sired by males (*ii*b) do not count for fitness. In these seeds the nuclear allele of the cosexual becomes permanently associated with the male cytoplasm. Males only produce males. The nuclear allele never returns to the neutral cytoplasm. Outcrossed seeds sired by male cytotypes are a sink. Second, for siring seeds on other cosexuals (*iii*a), pollen of the cosexuals now needs to compete with pollen from males, which reduces their chance of success. Only the outcrossed seeds that are sired by cosexuals (*ii*a and *iii*a) count towards fitness.

### Selfing Rating Increases with Male Allocation

All cosexuals together make $gr$ pollen. The males together make $1-g$ pollen. The chance that a seed is sired by cosexuals and contributes to fitness is $gr/(gr+1-g)$*.* There are *g* cosexuals left so divide by *g* to arrive at the fitness contribution per cosexual plant. The number of outcrossed seeds available is $gF(1-S)(1-r)$. Fitness consists of the selfed seeds (*i*), outcrossed seeds on the mother plant that are sired by cosexuals (*ii*a) and seeds sired on other cosexuals (*iii*a). Absolute fitness of a rare mutant with sex allocation *r*_*m*_ in a population in which *r* is the common strategy is:

10 $${W}_{m}=2(1-\delta ){S}_{m}(1-{r}_{m})+\frac{r}{gr+1-g}gF(1-{S}_{m})(1-{r}_{m})+\frac{r}{gr+1-g}gF(1-S)(1-r)\frac{{r}_{m}}{r}$$

Comparing with the simpler Eq. (2a) with only cosexuals shows that the multiplier *F* has now been replaced by $Fgr/(gr+1-g)$*,* which is smaller than *F*. The presence of cytoplasmic males reduces the fitness contribution of outcrossed seeds and its expected effect (compare Eq. 2b) is increased female allocation in the cosexuals. Results are complicated, however, by $d{S}_{m}/d{r}_{m}$ which can take on any (positive) value depending on the shape of the selfing curve. Also fitness minima are possible with high inbreeding depression (paragraph 2.4) The candidate ESS value for r* can still be calculated. For example, with only cosexuals in the population and $S={r}^{0.1}$, $\delta =0.4$ and *F* = 1, the population converged to r* = 0.155 with a selfing rate of 0.830. With 50% males r* = 0.099 was reached, a shift to more female allocation, with a slightly lower accompanying selfing rate of 0.793. Because of the complexity we stop here and focus on the model with constant selfing, which does allow analytical results. Note that the model could be extended even further by assuming that also fertilisation rate is not constant but increases with *r* and the fraction males in the population.

### Constant Selfing Rate

#### Constant Fertilisation Rate

We take selfing rate *S* constant in Eq. (10):

11a $${W}_{m}=2(1-\delta )S(1-{r}_{m})+\frac{r}{gr+1-g}gF(1-S)(1-{r}_{m})+\frac{r}{gr+1-g}gF(1-S)(1-r)\frac{{r}_{m}}{r}$$

Setting $d{w}_{m}/d{r}_{m}=0$ gives the candidate ESS. We write *p* for the function $p(\delta ,S)$:

11b $$r^{*}=\frac{gF-p+pg}{pg+2gF}$$

**Fig. 4:**
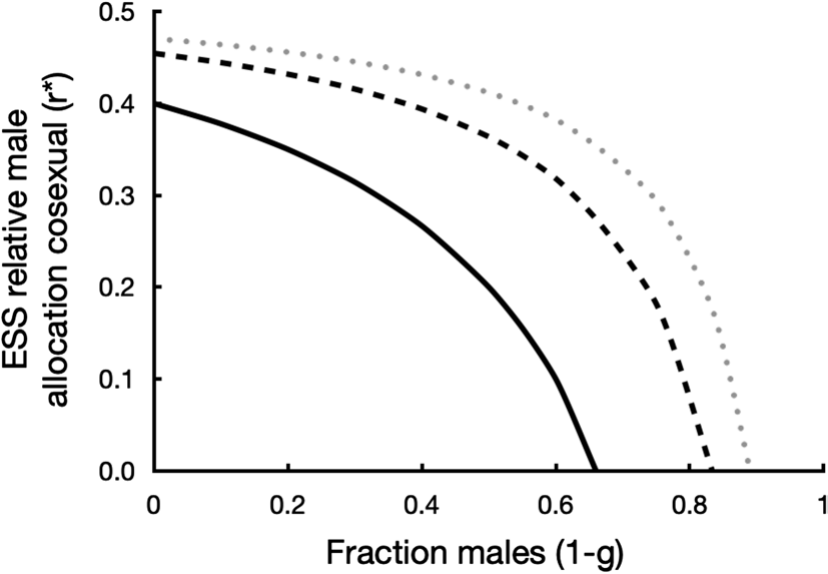
A fixed fraction cytoplasmic males (1-*g*) in the population selects for lower ESS allocation to male (r*) in cosexuals, i.e. for relatively more female allocation (Eq. 11b). The effect is strongest with a low fertilisation rate (*F*) (solid line *F* = 0.2, broken line *F* = 0.5, stippled line *F* = 0.8)

In fully outcrossing populations ($S=0$ and therefore $p=0$) the ESS is again equal allocation to male and female ($r^{*}=0.5$). The population converges to the ESS when ${d}^{2}{w}_{m}/d{r}^{2}>0$. With ${r}_{m}=r=r^{*}$ this yields ${d}^{2}{w}_{m}/d{r}^{2}=2F(1-S)({g}^{2}-r{g}^{2}+2rg-3g+2)/(gr+1-g{)}^{3}$. The sign of the second derivative depends on the term$({g}^{2}-r{g}^{2}+2rg-3g+2)$, which is always positive in the permitted range, between 0 and 1, of *g* and *r*. Because $dr^{*}/dg<0$, ESS male allocation always increases with frequency of cosexuals (*g*). Easier said, cosexuals are selected to allocate more to female in the presence of cytoplasmic males. The feminising effect is strongest with low fertilisation rates (Fig. 4). The effects of parameters *S*, $\delta$ and *F* on r* are the same as in the model without cytoplasmic males (Eq. 2b).

Under what conditions do cosexuals reach $r^{*}=0$ in this model? This can only be the case if the numerator in Eq. (11b) becomes zero, i.e. when $gF-p+pg=0$. This gives: $1-g\ge F/(F+p)$. When the frequency of males rises above the threshold $F/(F+p)$, cosexuals are selected to allocate only to female.

#### Fertilisation Rate Increases with Fraction Males in the Population

Alternatively, fertilisation rate is not fixed but increases with the amount of pollen produced. The cosexuals together produce $gr$ and the males $1-g$ pollen: $F=(gr+1-g{)}^{\alpha }$ and with $\alpha =1$ $F=gr+1-g$. Without males the fertilisation rate is *r.* When males dominate fertilisation approaches 1. When substituting this equation for *F* in Eq. (11a) the factor $gr+1-g$ cancels and we obtain:

12a $${W}_{m}=2(1-\delta )S(1-{r}_{m})+gr(1-S)(1-{r}_{m})+gr(1-S)(1-r)\frac{{r}_{m}}{r}$$

Fitness Eq. (12a) with cytoplasmic males is identical to Eq. (9a) with cytoplasmic females in the population. Again the population converges to the weak ESS r*:

12b $$r^{*}=0.5-0.5p(\delta ,S)/g$$

Equation 12b depends in a similar way on model parameters as Eq. 11b with fixed fertilisation rate. In Eq. 12b more than a fraction $1-p$ males in the population selects for $r^{*}=0$, i.e. cosexuals should allocate 100% to female. It may be surprising that adding cytoplasmic males to the population (Eq. 12b) has the same effect on sex allocation of the cosexual as adding cytoplasmic females (Eq. 9b). Fertilisation rate increases with the fraction males and decreases with the fraction females. Note, however, that for the males we took into account that only the outcrossed seeds sired by cosexuals (component *ii*a and *iii*a of fitness) count for fitness. In this case one could refer to factor $gr$ in Eq. 12a as the ‘effective’ fertilisation rate, the fraction of outcrossed seeds produced that actually contributes to fitness. Effective fertilisation declines with the fraction cytoplasmic males.

## Simultaneous Change of the Fraction Cosexuals and Sex Allocation (With Constant Selfing)

So far we assumed the fraction females (paragraph 4) or males (paragraph 5) was fixed and then calculated r*. Or we calculated the equilibrium fraction cosexuals for a fixed value of *r* (paragraph 3). But the two parameters may change simultaneously and this could possibly lead to runaway selection. The shift in frequency of the cosexual is rapid and due to competition between the neutral and male cytotype. The change in sex allocation of the cosexual concerns an evolutionary shift and depends on genetic variation in the population and mutations that generate new variation. ESS models typically assume that a mutant is slightly different from the common type. The mutant invades in a monomorphic population and replaces the common type. Then after some time a slightly different new mutant arises and so on, until the ESS is reached and no mutant can invade any more. With respect to sex allocation, one could start at $r=0$* a*nd then ask whether a mutant with ${r}_{m}=0.01$ could invade and take over*.* Next when $r=0.01$ has become the common type, can a mutant with ${r}_{m}=0.02$ invade and take over? And so on until we reach an ESS in which no mutant can invade. We use a similar idea here by starting at *r* = 0 and a corresponding equilibrium fraction females (Eq. 5) or males (Eqs. 6c, 7). Next we ask whether a mutant with ${r}_{m}=0.01$ can invade and take over. If yes, we move on to $r=0.01$*.* We then adjust the fraction females or males accordingly. Next we examine whether a mutant can establish in a population that has both $r=0.01$ and a new corresponding fraction males or females*.* With this procedure changes in sex allocation and fraction males or females follow each other with small steps. Two cases can be distinguished. First, the fitness gradient $d{w}_{m}/d{r}_{m}$ is positive in $r=0$. In this case an intermediate ESS exists for r*. Second $d{w}_{m}/d{r}_{m}$ is negative in $r=0$. Mutants with more male allocation cannot invade and $r^{*}=0$. We followed the population trajectory with simulation, keeping in mind that $\tilde{g}$ is always between 0 and 1. Once the population reaches $\tilde{g}=1$ females or males are eliminated and we return to the situation with cosexuals only.

### With Female Cytotypes

The only interesting case here is when fertilisation decreases with the fraction females (paragraph 4.2). The fitness gradient (Eq. 9a) in $r=0$ is:

13a $$d{w}_{m}/d{r}_{m}=-2(1-\delta )S+\tilde{g}(1-S)$$

With $\tilde{g}=1$ (only cosexuals in the population) this gives the same results as in paragraph 2.5: an intermediate ESS exists when $(3-2\delta )S<1$, i.e. with low selfing rates and high inbreeding depression. Note from Eq. (13a) that the term on the left hand side is negative and the term on the right hand side is positive. Less cosexuals and more females always result in more cases in which $d{w}_{m}/d{r}_{m}<0$, corresponding to $r^{*}=0$. With $r^{*}=0$ cosexuals no longer export pollen, females are not fertilised and disappear. The more realistic case is the intermediate ESS. Imagine starting at $r=0$ and moving in the direction of more male allocation. A mutant with ${r}_{m}=0.01$ can invade and take over the population, which then consists fully of individuals with $r=0.01$. From Eq. (5) it followed that $d\tilde{g}/dr<0$. More male allocation in the cosexual, 0.01 instead of 0, selects for a lower fraction cosexuals and more females. An increase of cytoplasmic females in the population selects for less male allocation (Eq. 9b), i.e. pushes the population back in the direction from which it came. With this counterforce an ESS can exist at an intermediate value of r*, that can be found by solving Eqs. (5) and (9b):

13b $$r^{*}=(1-S)/(3-S)$$

Simulations showed that the population always converged (from starting point $r=0$ or $r=0.5$) to this weak ESS, provided that females persisted ($\tilde{g}<1$). With full outcrossing ($S=0$) of course no cosexuals remain (Eq. 5). Equation 13b then states that when *S* approaches 0, ESS male allocation is close to 1/3. The mostly outcrossing population is then gynodioecious with many females and few cosexuals that allocate r* (Eq. 13b) to pollen.

### With Male Cytotypes

With constant fertilisation the fitness gradient in $r=0$ is $d{w}_{m}/d{r}_{m}=-(1-\delta )S$ (using Eq. 11a with constant *S* and Eq. (6c) so that the fraction cosexuals $\tilde{g}$ is at equilibrium). This fitness gradient is always negative. When we start at $r=0$ a mutant with ${r}_{m}=0.01$ cannot invade the population. Apparently at the equilibrium $\tilde{g}$ corresponding to $r=0$, there are already so many males and so few opportunities to sire seeds on other cosexuals in the population that it is not profitable for the cosexual to invest any resource in male. This is the case even for parameters for which Eq. (9b) predicted an intermediate ESS, under the assumption that the fraction males in the population was fixed. With males in the population $d\tilde{g}/dr>0$, which is opposite to the situation with females in paragraph 6.1. When we started simulations at $r^{*}=0.5$, successive mutants with lower *r*-values established because selfing selects for female bias in the cosexual. The population reaches $r=0.49$, but as soon as this occurs the fraction males increases. These extra males select for even more female allocation, which selects for more males, and so on until r* = 0 is reached. As a result of this self-reinforcing effect, in the end the population always consists of males and cosexuals that allocate fully to female ($r^{*}=0$) while retaining some selfing (*S* was fixed in the simulations). One could refer to this scenario as runaway selection.

We also examined the case in which fertilisation rate was not fixed but increased with pollen in the population ($F=gr+1-g$). In $r=0$, $d{w}_{m}/d{r}_{m}=-(1-\delta )S$ (using Eq. 12a and substituting the value for $\tilde{g}$ from Eq. 7) which is again always negative. Also in this case $r^{*}=0$ was always the ESS.

In conclusion to paragraphs 4, 5 and 6, only the presence of cytoplasmic males leads to sexual specialisation. With males in the population, cosexuals evolve towards 100% allocation to female ($r^{*}=0$*)* by runaway selection. We refer to this final situation as subdioecy and it seems we are close to dioecy. A mutation with the effect that cosexuals loose their selfing ability, would lead to dioecy with cytoplasmic males and nuclear females. However, this is an unstable situation. Without selfing, female-biased cosexuals do not pass on their neutral cytoplasm to the next generation. A mutation that eliminates selfing would disappear immediately from the population. To understand how the transition from subdioecy to dioecy may occur we need to consider genetic models in the next paragraph 7.

## A genetic Model with a Male Cytotype and a Nuclear Gene that Switches Sex

After sexual specialisation, what happens when an allele arrives that changes sex of the cytoplasmic males? Does this lead to loss of the neutral cytoplasm and dioecy? Or can cosexuals maintain themselves? We examine two cases. First full sexual specialisation, cosexuals have selfing but are not exporting pollen ($r=0$). Second less specialisation, cosexuals retain some pollen production ($r=0.2$). In both cases initial populations consist of cosexuals and male cytotypes.

### Males, Females and Partially Selfing Cosexuals with *r* = 0 in One Population

**Fig. 5:**
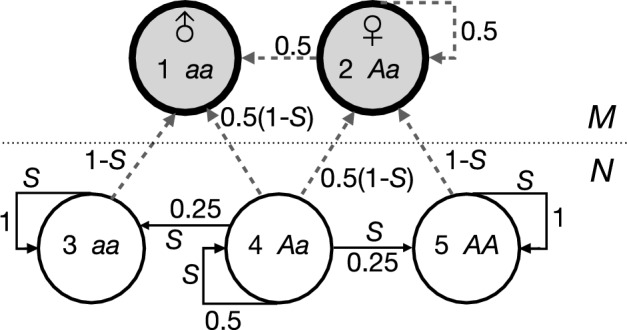
Generation of different genotypes by seed production when cosexuals (4–6, neutral *N-*cytotype) allocate fully to female (*r* = 0) while retaining some selfing (see also Appendix 1). CytDNA is paternally inherited through the pollen. Note that the flow of resources through seed production is from the neutral *N*-cytotype (type 4 to 6, open circles) to the male *M*-cytotype (type 1 and 2, shaded circles)

What happens when a nuclear allele arrives that counters the effect of the male cytoplasmic factor and changes a male into a female? We use a genetic model that starts from subdioecy, male cytotypes and female-biased cosexuals together. In these populations the *r* = 0 allele is present in all plants. Males have cytotype *M*, cosexuals have the neutral *N* cytotype. Consider a nuclear gene with two alleles. The recessive allele *a* has no effect on the phenotype. Males are *aa/M*. The dominant allele *A* arises by mutation. It fully counteracts effects of the male cytotype *M* and switches sex: *AA/M* and *Aa/M* genotypes become fully female without selfing. Allele *A* does not affect the phenotype of the cosexual with the neutral cytotype *N*. With one nuclear gene with two alleles (*A* and *a)* and two cytotypes (*M* and *N*) six genotypes are possible (Appendix). The calculation of the genotype frequencies and starting conditions are given in the Appendix. Figure 5 illustrates seed production of possible crosses.

**Fig. 6:**
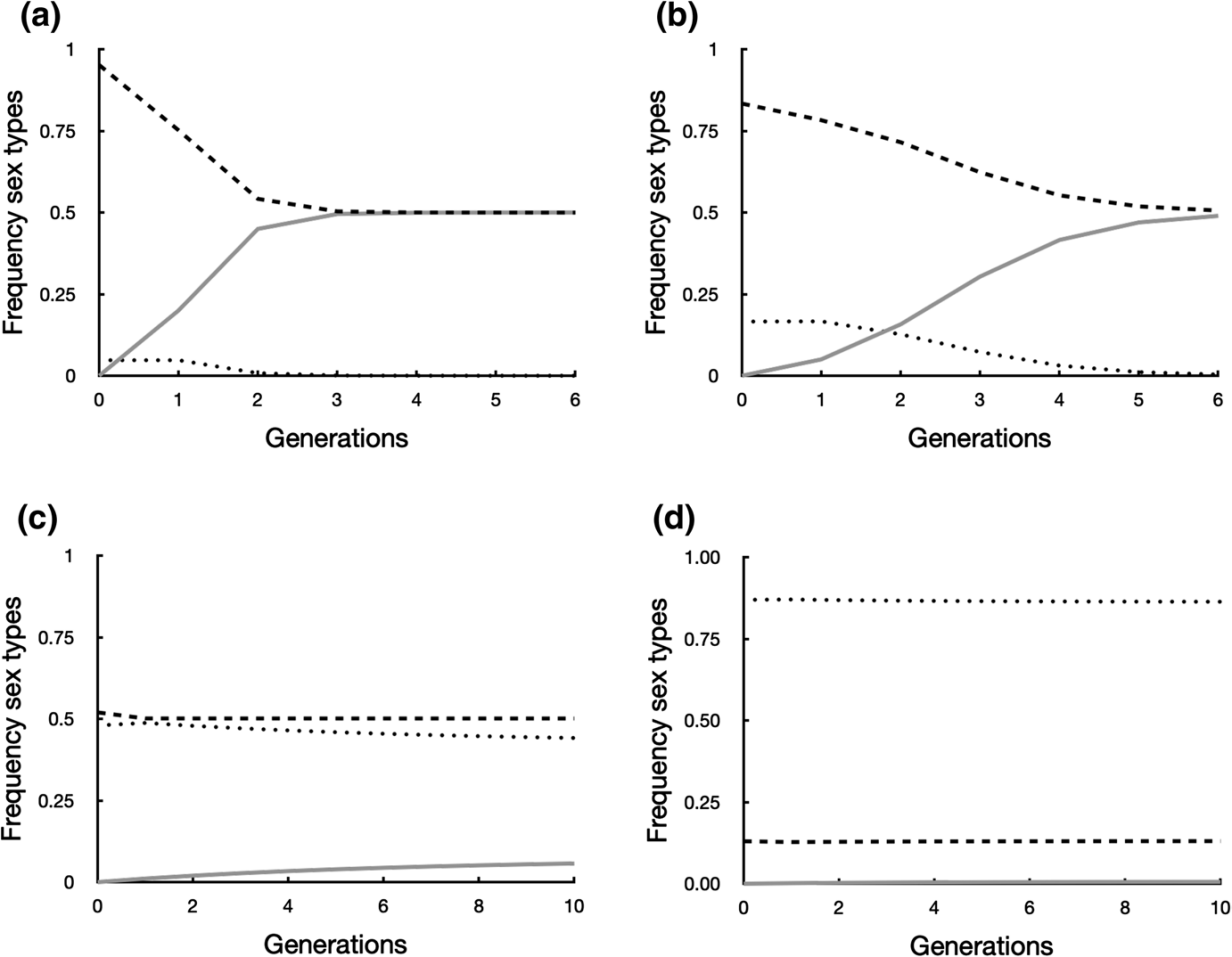
Frequencies of males (broken line), cosexuals (stippled line) and females (solid line) change stepwise in successive generations after introduction at time 0 of a dominant allele *A* at 1% frequency. Allele *A* changes an *aa/M* male into an *Aa/M* female. At the time of introduction the population was at the equilibrium frequency predicted by Eq. (6c) and consisted of males (*aa/M*) and cosexuals (neutral cytotype *N*) that allocate all resources to female (*r* = 0) while retaining some selfing. **a** The *A*-allele rapidly sweeps through the population, leading to dioecy with 50% *Aa/M* females and 50% *aa/M* males and rapid loss of the cosexuals with the *N-*cytotype with the parameters *S* = 0.2, $\delta =0.8$ and *F* = 1. **b** Even with low inbreeding depression ($\delta =0.2$) and other parameters the same (*S* = 0.2, *F* = 1) the cosexual with the *N*-cytotype is rapidly lost from the population. **c** At more favourable conditions for the cosexual (*S* = 0.4, $\delta =0.5$, *F* = 0.35) it is maintained in the population next to males and females. **d** With even more favourable condition for the cosexual (*S* = 0.9, $\delta =0.4$, *F* = 0.8) they dominate the population, but males and very few females (1%) persist

**Fig. 7:**
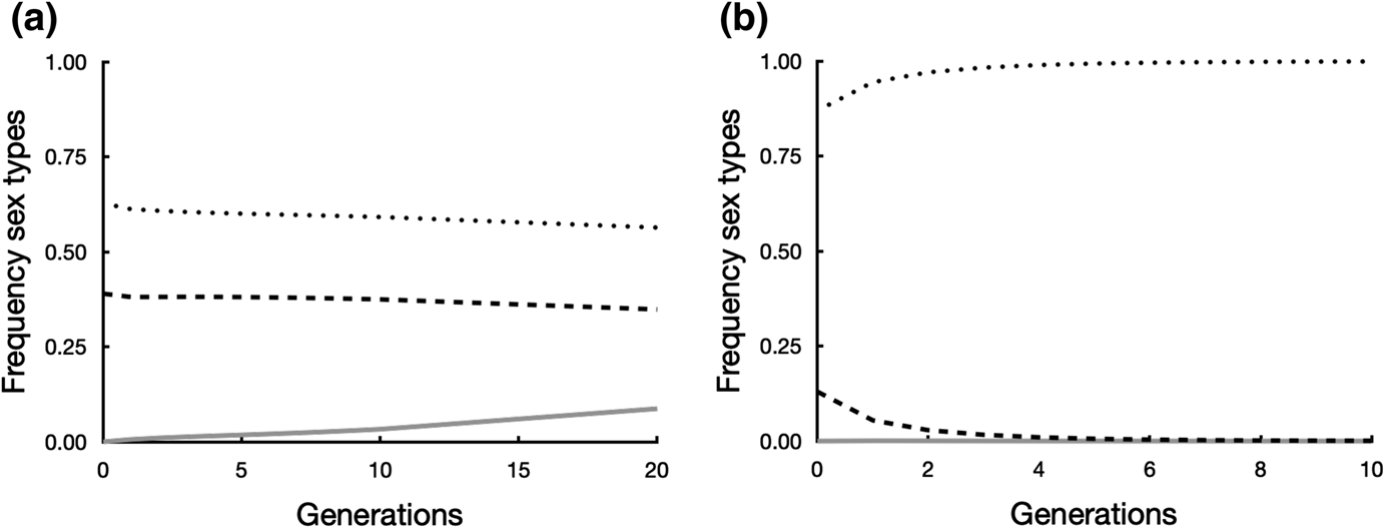
Frequencies of males (broken line), cosexuals (stippled line) and females (solid line) change stepwise in successive generations after introduction at time 0 of a dominant allele *A* at 1% frequency. Allele *A* changes an *aa/M* male into an *Aa/M* female. At the time of introduction the population was at the equilibrium frequency predicted by Eq. (6c) and consisted of males (*aa/M*) and cosexuals (neutral cytotype *N*) that still allocate some resources to male (*r* = 0.2) and are partially selfing. **a** More cosexuals are now maintained in the population next to males and females with the same parameters as in Fig. 6c (*S* = 0.4, $\delta =0.5$, *F* = 0.35). **b** Cosexuals now rapidly expel both males and females from the population with the same conditions as used in Fig. 6d (*S* = 0.9, $\delta =0.4$, *F* = 0.8)

With parameters that may be typical of conifers (*S* = 0.2 and $\delta =0.8$) and *F* = 1, the dominant *A*-allele sweeps through the male-biased population in just three steps (Fig. 6a). After three generations cosexuals with the *N-*cytotype were already lost and only the *M*-cytotype remained. Only males (genotype 1) and females (genotype 2) remained (see Appendix), both at frequency 50%. This system has nuclear sex determination and 50% *Aa/M* females and 50% *aa/M* males. This transition from monoecy to dioecy is reversible: if conditions change so that they greatly favour the cosexual (*F* = 0.8, *S* = 0.9, $\delta =0.4$ as in Figs. 6d and 7b) and a neutral cytoplasm is introduced into the dioecious population, cosexuals will establish and eventually coexist with 18% males and 18% females (results not shown).

Why are cosexuals performing so poorly under the conditions of Fig. 6a? Cosexuals pass on their *N*-cytoplasm to the next generation only through selfing (Fig. 5). All investment in outcrossed seeds is wasted from the point of view of the *N*-cytotype, since all these seeds are sired by the *aa/M* male and will then contain the paternal *M-*cytotype. With the parameters from Fig. 6a, S = 0.2 and $\delta =0.8$, there are no fitness returns from making outcrossed seeds. These outcrossed seeds make up 80% of all resources invested in seeds. Selfed seeds have only 20% of the viability of outcrossed seeds. Together these two factors reduce fitness of the cosexual by a factor 25.

Even with low inbreeding depression ($\delta =0.2$, Fig. 6b) the cosexual is rapidly lost and dioecy evolves after only six generations. This is a surprising result because in the classic models dioecy evolves when inbreeding depression is high ($\delta >0.5$), not when it is low (de Jong and Klinkhamer 2005). The reason for the rapid loss of the cosexual is the same as in Fig. 6a, with *S* = 0.2 there are no fitness returns for the investment in outcrossed seeds, which make up 80% of all seeds produced. Additional simulations showed that the final outcome in Fig. 6a, b, dioecy with 50% males and 50% females, does not depend on starting frequencies. As long as the *A*-allele is present somewhere in the population, dioecy was reached with 50% males and 50% females.

Can cosexuals maintain themselves when conditions favour reproductive assurance? Cosexuals benefit from high selfing rate, low inbreeding depression and low fertilisation rate. Under such conditions coexistence of all three types (males, females, cosexuals) was observed in simulations. In the example in Fig. 6c we observed after 10 generations 44% cosexuals, 50% males and only 6% females. When conditions become even more favourable for the cosexual (Fig. 6d) they dominated (86% after 10 generations) but coexisted with 13% males and 1% females. Males and females persisted even when conditions greatly favoured cosexuals. This occurs because, in this version of the model, resources flow from cosexuals to males and females but not in the other direction (Fig. 5). As long as cosexuals are outcrossing both males and females continue to be generated.

The simulations results in Figs. 6c and d, depended on initial genotype frequencies and did not necessarily lead to equal numbers of males and females. The reason for this is simply that we started with more of allele *a* than of allele *A*. Repeated selfing leads to rapid loss of the *Aa/N* cosexuals while *AA/N* and *aa/N* persist through selfing and have equal fitness. Genotype *aa/N* is then most common from the start on. When fertilised by an *aa/M* male this cosexual produces 100% *aa/M* (male) seeds. The rarer *AA/N* cosexual produces 100% *Aa/M* (female) seeds when fertilised by the same male.

### Males, Females and Partially Selfing Cosexuals with *r* = 0.2 in One Population

What happens when the *A*-allele arrives in a population with less sexual specialisation? We arbitrarily chose *r* = 0.2 for the cosexual. This is not the ESS value but still the corresponding equilibrium starting frequency of males follows from Eq. (6c). The cosexuals with $r=0.2$ export some pollen and also pass on their *N*-cytotype by siring outcrossed seeds on other cosexuals and on females with the *M*-cytotype. This results in a flow of resources from females to cosexuals, which is an improvement for cosexuals as compared to $r=0$, as in Fig. 5.

With the parameters from Fig. 6a, b we obtained the same result as in paragraph 7.1 The cosexual with the *N*-cytotype was eliminated and dioecy obtained, but at a slower rate as before (results not shown). The transition from monoecy to dioecy was again reversible when conditions changed in favour of the cosexual (results now shown).

With the parameters from Fig. 6c that favoured cosexuals, coexistence of the three types was again possible. The population now contained after 10 generations 59% cosexuals with *r* = 0.2 (Fig. 7a), instead of 44% cosexuals with *r* = 0 (Fig. 6c). When conditions became even more favourable for the cosexual, males and females disappeared from the population, regardless of initial genotype frequencies (Fig. 7b). The loss of males is in line with the prediction from Eq. (6d).

In summary of paragraph 7, simulations illustrate that a nuclear allele that switches sex can establish in a population consisting of cytoplasmic males and female-biased cosexuals with partial selfing. This leads to loss of the neutral cytoplasm and dioecy, or, under conditions that favour reproductive assurance (low $\delta$ and *F*, high *S*), to populations in which cosexuals either dominate or coexist with both males and females.

## Discussion

### Gynodioecy Versus Androdioecy

Gynodioecy has been extensively studied in angiosperms as a likely first step on the pathway towards dioecy. Yet the importance of this route is currently under debate. Based on the co-occurrence of gynodioecy and dioecy in the angiosperm phylogeny, Dufay et al. (2014) considered this the major route to dioecy. A review by Spigler and Ashman (2012) raised some questions. Renner and Müller (2021) concluded that the gynodioecy pathway is only important in the genus *Silene*. In the simple models analysed in this paper the presence of cytoplasmic females does not automatically induce selection for more male allocation in cosexuals. This presents a problem because restorer genes or genes that switch sex are more likely to be come established in populations with sexual specialisation (Schultz 1994). Of course, alternatives can be analysed. We assumed prior selfing, but outcross and self pollen grains could also compete for access to ovules. In that case the presence of cytoplasmic females would lower the amount of dispersing pollen in the population and this would then lead to higher selfing rates. Higher selfing rates always select for more female allocation in cosexuals. So this alternative scenario leads away from sexual specialisation in gynodioecious species.

More complex genetic models of gynodioecy with one or more restorer alleles present do result in sexual specialisation. The cytoplasmic compartments are then no longer isolated, female cytotypes are no longer just a sink and nuclear genes can flow from one compartment to another. In this scenario cosexuals are selected to become more male in the presence of cytoplasmic females (Maurice et al. 1993, 1994) and sexual specialisation increases the probability that the neutral cytotype is lost (Maurice et al. 1993). A sex-switching allele may then invade and lead to dioecy with full nuclear control over gender (Schultz 1994). In a later stage the “default” feminising factor could move from the cytoplasm to the nucleus since such gene transfer is a common phenomenon in plants (Brandvain and Wade 2009). This scenario for the transition from gynodioecy to dioecy is realistic and plausible. However, whether sexual specialisation can be found in gynodioecious species in nature remains an issue. Several authors found no correlation between frequency of females and sex allocation of cosexuals when comparing different field populations (Spigler and Ashman 2012).

With paternal inheritance of cytDNA sexual specialisation is rapid in our models and the unexplored route, from monoecy via cytoplasmic androdioecy to dioecy, seems plausible. Of course, dioecy could evolve from selection on nuclear sex allocation genes only, without the involvement of cytoplasmic genes. The model in paragraph 2.4 suggests that this could occur gradually when inbreeding depression is high, selfing rate increases with male allocation and outcrossing is easy (high fertilisation rate and low costs of unfertilised female cones). While the first condition will be met in most conifer species, this is not obvious for the other conditions. For rare conifer species with low fertilisation rates female cost may well be large and monoecy may still be the ESS. Also these conditions for the evolution of dioecy are general, apply to all seed plants, and are unlikely to explain the difference between large families like the Podocarpaceae (94.9% dioecy) and Pinaceae (0% dioecy, Fig. 1). This difference rather suggest that some major factor is different between these groups. We argued that the different mode of mtDNA inheritance is a relevant factor to consider when comparing reproductive systems of plants.

Paternal transfer of cytDNA is rare in angiosperms and only three cases are known. Melons and cucumbers in the dioecious *Cucumis* genus ( Havey et al. 1998; Zhang et al. 2006) and bananas (*Musa*, monoecious, Fauré et al. 1994) show paternal transfer of mtDNA and maternal transfer of cpDNA. In the kiwifruit family (Actinidia, dioecious) mtDNA transfer is maternal and cpDNA paternal (Chat et al. 1999). No doubt the paternal transfer of cytDNA was discovered because these three species are of commercial importance and were used in breeding programs. It would be interesting to know the mode of inheritance of cytDNA for more angiosperms and its possible association with dioecy.

### Different Assumptions About the Interaction Between Cytoplasmic and Nuclear Genes

Restorer genes in angiosperms are as diverse as CMS types (Hu et al. 2014). They could work specifically against one cytotype or more general against several CMS types. In the genetic model we assumed that the dominant *A-*allele switched sex of the *aa/M* male to an *aA/M* female, without affecting the phenotype of the cosexual. Different assumptions could be made here. A typical restorer gene would change the *aa/M* male into a cosexual with either *r* = 0 (paragraph 7.1) or *r* = 0.2 (paragraph 7.2). A dominant nuclear allele with strictly a strong feminising effect, overruling all male factors, would render all plants in which it resides female. Females would then occur next to *aa/M* males and aa*/N* cosexuals (Table 1). These and other alternatives (summarised in Spigler and Ashman 2012) can be explored by further simulations, but is beyond the scope of this paper. In our simulations the cosexual had a large disadvantage in a mostly outcrossing population and the neutral cytoplasm was rapidly eliminated. It is likely that this elimination of the neutral cytotype also occurs in variations on the same theme (Table 1).

**Table 1:** Alternative assumptions about how the dominant nuclear allele *A* leads to different phenotypes of plants with the male (*M*) or neutral (*N*) cytotype

Genotype	Sex switch gene	Restorer gene	Feminising gene
*aa/M*	Male	Male	Male
*Aa/M*	Female	Cosexual with selfing	Female
*AA/M*	Not formed	Cosexual with selfing	Not formed
a*a/N*	Cosexual with selfing	Cosexual with selfing	Cosexual with selfing
*Aa/N*	Cosexual with selfing	Cosexual with selfing	Female
*AA/N*	Cosexual with selfing	Cosexual with selfing	Female

### Do Cytoplasmic Genes Effect Sex Allocation in Conifers?

Mitochondria in the Pinaceae are large (5.9 Mb in *Picea glauca*, as compared to the 0.2–2 Mb range for angiosperms), variable and contain many genes with unknown function (Neale and Wheeler 2019). Chloroplasts of conifers and angiosperms are of similar size (0.12–0.17 Mb, Neale and Wheeler 2019). Mitochondria of gymnosperms seem to have as much potential as those of angiosperms to interfere with sex allocation. This could be through male or female sterility or by changing gender of the cone at initiation. Yet there appears to be no direct evidence for the role of cytoplasmic factors in determining gender in conifers. Reciprocal crosses can unequivocally show effects of cytoplasmic genes when characters of the two *F*_1_’s differ (Koelewijn and van Damme 1995). Such reciprocal crosses have been performed in conifers but focussed on agronomic characters of young plants and, to our knowledge, not on reproductive characters of parents and offspring. Long generation time of conifers is also an issue. It is perhaps possible to investigate already established and reproducing *F*_1_ trees from reciprocal crosses that were made in breeding programs or occurred naturally in hybridisation zones between species. If one species has a feminising or masculinising cytoplasm that is to some extent restored and the other species lacks these genes, this would immediately show from the different gender of the two *F*_1_’s. This would be a strong suggestion that cytoplasmic factors are involved in determining gender. No doubt knowledge about effects of environmental factors, hormones and genes on cone identity in conifers will advance in the coming decades. In these research programs the potential effects of mtDNA and cpDNA on gender should be considered.

### Field Data on Sex Allocation in Monoecious Conifers

When monoecious plants maintain themselves in the population, they will often occur together with both male and female plants (Figs. 6c, 7a). This is a rather unexpected prediction from the genetic model. Some authors refer to polygamy or trioecy and this system is very rare in angiosperms (Renner 2014). Does this pattern occur in conifers in the field?

Documenting such patterns is complicated by effects of environment (Freeman et al. 1981) or plant age on gender. For instance, some *Pinus* species start reproduction as female and only later produce male cones also, making older trees monoecious (Shmida et al. 2000). Other plants change sex with all possible transitions between male, monoecious and female being observed (Vasek 1966; Allison 1991; Jordano 1991; Arista and Talavera 1997). Nevertheless some authors found no or weak effects of size or age (Allison 1991; Jordano 1991; Flores-Renteria et al. 2013) and a strong genetic component to gender. Nine studies quantified gender in conifers listed as monoecious by Walas et al. (2018) (Table 2) and all studies documented trioecy. In addition to Table 2, populations of *Juniperus excelsa* in Lebanon were almost completely dioecious with equal proportions of males and females (Douaihy et al. 2013) when they grew above 2200 m. At intermediate altitudes monoecious plants dominated (62–93%), sometimes accompanied by females (2 populations), males (1 population) or both (1 population). Also monoecious individuals of the species in Table 2 showed a range of phenotypic gender. This is unlike the typical pattern in cosexual angiosperms, that show limited variation around a mean value for phenotypic femaleness (Lloyd 1980). With maternal mtDNA transfer one expects a female bias in populations and with paternal transfer a male bias. Females are indeed more common than males in the Pinaceae and less common in the Taxaceae (Table 2), but differences are small and data are few. In various *Juniperus* populations females tended to be more common than males, which is not expected with paternal mtDNA inheritance. Effects of mode of inheritance of mtDNA on plant sex systems should further be explored in conifers. In order to make progress, it would be pivotal to know with certainty the mode of mtDNA inheritance for individual species, especially in the diverse Cupressaceae family, instead of inferring this mode from other species in the same genus or family.

**Table 2:** Occurrence of cosexual (monoecious), male and female individuals in natural populations of conifer species listed as monoecious by Walas et al. (2018)

Species	Family	mtDNA transfer^1^	%cosexual	%male^2^	%female	Author
*Abies pinsapo*	Pinaceae	Mat	9	16	75	Arista and Talavera (1997)
*Pinus johannis*	Pinaceae	Mat	1	40	59	Flores-Renteria et al. (2013)
*Cedrus deodara*	Pinaceae	Mat	48.4	13.6	38.0	Khanduri et al. (2021)
*Juniperus australis*	Cupressaceae	Pat	4.4	46.1	49.5	Vasek (1966)
*J. californica*	Cupressaceae	Pat	1.8	42.6	55.6	Vasek (1966)
*J. occidentalis*^*3*^	Cupressaceae	Pat	51.5	10.2	38.3	Vasek (1966)
*J. osteosperma*	Cupressaceae	Pat	89.2	5.1	5.7	Vasek (1966)
*J. phoenicea*^*4*^	Cupressaceae	Pat	40–50	< 15	31–40	Jordano (1991)
*Taxus canadensis*	Taxaceae	Pat	64–79	17–30	4–6	Allison (1991)

^1^*mat *maternal, *pat *paternal, not known for these species but based on observations on other species in the same genus or family

^2^Individuals that were almost completely male were lumped with those that were fully male. The same procedure was followed for the females

^3^Total of 3 populations

^4^Estimates refer to Spanish populations. Moroccan populations consisted almost completely of monoecious plants with gender varying continuously between male and female

Applying the label “monoecy” to individual plants is straightforward. But denoting species as monoecious is confusing, as it may falsely suggest that all plants in the population have the same sex-allocation strategy. Surprisingly, all studies showed populations containing a mix of monoecious, male and female individuals in various proportions (Table 2). The classification of *Pinus johannis* as monoecious (Walas et al 2018) is peculiar considering the data in Table 2. Botanical terminology may lead away from asking interesting questions about gender distributions in conifer populations. In *Juniperus* section Sabina some species are listed as dioecious. Yet Adams (2014) commented: “The apparent ease with which male *Juniperus arizonica* plants appear to produce a few female cones seems to indicate the dioecious/ monoecious mode is somewhat porous and may be easy to bridge.” Species, denoted as “monoecious” are apparently a mix of male, female and monoecious individuals in varying proportions (Table 2). The appropriate question in *Juniperus* is: “Under what conditions do monoecious individuals maintain themselves in populations?” In the models in this paper monoecious plants benefit from reproductive insurance. This benefit is highest with high selfing, low inbreeding depression and low fertilisation rate and under these conditions monoecious individuals are expected to persist. This hypothesis can be tested. Low population density could be an indicator of low fertilisation. Junipers often grow on under a range of conditions. For instance, *J. tibetica* is native to the mountains of central China where it grows from 2700 to 4800 m altitude (Adams 2014). It would be possible to compare populations with different densities and ask whether monoecious individuals are most abundant at the marginal, low-density sites at the edge of its distribution. Douaihy et al. (2013) recorded, apart from altitude, also the density of *J. excelsa* trees, which ranged from 45 to 147 trees per ha. Population density was lowest at the high altitudes where the species was dioecious. This goes against the reproductive assurance hypothesis. Nevertheless this is a good way to test, and potentially reject, different hypotheses. Of course density is a rather indirect measure of *F* and more direct measures*,* such as pollen density, fertilisation rate and seed set are preferable. Interestingly densities of airborne pollen can be higher in the lower atmosphere then at ground level (Comtois et al. 2000) so that populations at high latitude may benefit from a greater inflow of pollen from distant populations. The approach of connecting the frequency of cosexuals to environmental and plant factors is more promising than calculating associations between various characters and sex system, classified as dioecy/monoecy.

## Concluding Remarks

The modeIs detailed how paternal transfer of mtDNA can lead to dioecy. It is encouraging that dioecy occurs in the family Taxaceae and genus *Juniperus* (both paternal mtDNA transfer) and does not occur in the Pinaceae (maternal mtDNA transfer). However, one should be careful in drawing this conclusion. There is not a single species for which detailed data on gender distribution in natural, undisturbed populations and mode of mtDNA inheritance are both available.

Mitochondrial inheritance may follow plastid inheritance, as Mogensen (1996) suggested, in which case mtDNA inheritance is expected to be paternal in most conifers. Or variation exists between species. In reciprocal crosses between *Pinus mugo* and *P. sylvestris* cpDNA inheritance was paternal in one direction and maternal in the other direction (Kormutak et al. 2018). A recent study (Chung et al. 2023) showed that chilling changed the mode of transmission of cpDNA from maternal to biparental in *Nicotiana tabacum*. Burt and Trivers (2006) suggested that cytoplasmic gynodioecy could select for a change of the mode of mitochondrial inheritance from maternal to paternal. The egg cell delivers many times more mitochondria and chloroplasts to the zygote than the sperm cell, so maternal inheritance is expected (Mogensen 1996). Studies on paternal inheritance suggested that maternal mitochondria are actively eliminated before or in the earliest stage of embryo development (Mogensen 1996; Burt and Trivers 2006). In the green alga *Chlamydomonas* maternal mtDNA was already completely eliminated during meiosis (Aoyama et al. 2006). Mutations that counteract this elimination process are conceivable. These new papers challenge the paradigm that the mode of cytoplasmic inheritance is a conservative character that rarely changes in evolution and is the same for all species in a genus or family.

Early studies used microscopic methods and fluorescent staining of maternal and paternal cytDNA in the earliest stage of embryo development. While these methods demonstrated elimination of maternal cytDNA, Mogensen (1996) argued that we cannot be sure that this process is complete and some maternal mitochondria or chloroplasts may catch on later. Modern genetic methods using polymorphic mtDNA markers can provide a definite answer (Mogensen 1996) and fill in the gaps in Fig. 1. For this purpose it is useful that many rare conifers already grow and reproduce in nearby botanical gardens and pinetums. Knowing the “hows and whys” (Mogensen 1996) of uniparental cytoplasmic inheritance is an interesting evolutionary question in its own right (Munasinghe and Ågren (2023) and conifers seem an excellent study system. Many conifers are vulnerable, threatened in their natural populations or are relicts from families that dominated the world millions of years ago. Conifers are now confined to specific habitats. It is important and urgent to collect more detailed data on their ecology and sex systems in these remaining natural habitats.

## Data Availability

Not applicable.

## Appendix

### Genetic model for two cytotypes (neutral and male) and a nuclear gene that switches sex of the male cytotype

The *N* cytotype is neutral and does not affect sex allocation, the *M-*cytotype is male. A dominant

allele *A* switches sex in the *M*-cytotype so that an *aa/M* male becomes an *Aa/M* female. Allele *A* has no effect in cosexuals. After introduction of the *A*-allele, the population consists of 6 genotypes: 1) *aa/M* males, 2) *Aa/M* females, 3) *AA/M* females, 4) *aa/N* cosexuals, 5) *Aa/N* cosexuals, 6) *AA/N* cosexuals, with corresponding frequencies ${f}_{1}..{f}_{6}$. Cytoplasms *N* and *M* are both paternally transmitted. Females produce *F* outcrossed seeds. Cosexuals produce $Q=F(1-r)(1-S)$ outcrossed seeds plus $(1-r)S$ selfed seeds that count for $1-\delta$ in the calculation of genotype frequencies. Pollen cost of selfing is negligible. Pollen is produced by males (*r* = 1) and the three cosexual genotypes (*r*), which totals $P={f}_{1}+r({f}_{4}+{f}_{5}+{f}_{6})$ pollen units in the population. This pollen is well-mixed and each genotype sires outcrossed seeds in proportion to the amount of pollen produced. In the equations we follow all seeds produced and by which type they were sired. For instance, ${f}_{4}Q({f}_{4}r/P)$ reads as: together all cosexuals of type 4 produce ${f}_{4}Q$ outcrossed seeds of which a fraction $({f}_{4}r/P)$ is sired by other cosexuals of type 4. The *AA/M* female (type 3) is never formed so its frequency is 0.

$${f}_{1}^{\prime}=0.5{f}_{2}F({f}_{1}/P)+{f}_{4}Q({f}_{1}/P)+0.5{f}_{5}Q({f}_{1}/P)$$

$${f}_{2}^{\prime}=0.5{f}_{2}F({f}_{1}/P)+0.5{f}_{5}Q({f}_{1}/P)+{f}_{6}Q({f}_{1}/P)$$

$${f}_{3}^{\prime}=0$$

$${f}_{4}^{\prime}={f}_{4}(1-\delta )S(1-r)+0.25{f}_{5}(1-\delta )S(1-r)+0.5{f}_{2}F({f}_{4}r/P)+0.25{f}_{2}F({f}_{5}r/P)+{f}_{4}Q({f}_{4}r/P)+0.5{f}_{4}Q({f}_{5}r/P)+0.5{f}_{5}Q({f}_{4}r/P)+0.25{f}_{5}Q({f}_{5}r/P)$$

$${f}_{5}^{\prime}=0.5{f}_{5}(1-\delta )S(1-r)+0.5{f}_{2}F({f}_{4}r/P)+0.25{f}_{2}F({f}_{5}r/P)+0.5{f}_{2}F({f}_{6}/P)+0.5{f}_{4}Q({f}_{5}r/P)+{f}_{4}Q({f}_{6}r/P)+0.5{f}_{5}Q({f}_{4}r/P)+0.5{f}_{5}Q({f}_{5}r/P)+0.5{f}_{5}Q({f}_{6}r/P)+{f}_{6}Q({f}_{4}r/P)+0.5{f}_{6}Q({f}_{5}r/P)$$

$${f}_{6}^{\prime}={f}_{6}(1-\delta )S(1-r)+0.25{f}_{5}S(1-\delta )(1-r)+0.5{f}_{2}F({f}_{6}r/P)+0.25{f}_{2}F({f}_{5}r/P)+{f}_{6}Q({f}_{6}r/P)+0.5{f}_{6}Q({f}_{5}r/P)+0.5{f}_{5}Q({f}_{6}r/P)+0.25{f}_{5}Q({f}_{5}r/P)$$.

The sum of the new frequencies is $T={f}_{1}^{\prime}+{f}_{2}^{\prime}+{f}_{3}^{\prime}+{f}_{4}^{\prime}+{f}_{5}^{\prime}+{f}_{6}^{\prime}$. In calculations new frequencies were then normalised by dividing ${f}_{1}^{\prime}..{f}_{6}^{\prime}$ by the common denominator *T* to ensure that their sum is always 1. All simulations started with *aa/M* males (*f*_1_) and *aa/N* cosexuals (*f*_4_) in the equilibrium frequency correctly predicted by Eq. (6c). At time 0 the dominant restorer allele *A* is then introduced at low frequency (1%) in the population in the heterozygote cosexual type 5 (*f*_5_ = 0.02 and *f*_2_ = 0, *f*_3_ = 0, *f*_6_ = 0).
